# The Genomic Landscape of Thyroid Cancer Tumourigenesis and Implications for Immunotherapy

**DOI:** 10.3390/cells10051082

**Published:** 2021-05-01

**Authors:** Amandeep Singh, Jeehoon Ham, Joseph William Po, Navin Niles, Tara Roberts, Cheok Soon Lee

**Affiliations:** 1 Discipline of Pathology, School of Medicine, Western Sydney University, Campbelltown 2560, Australia; 19202844@student.westernsydney.edu.au (J.H.); navinniles@me.com (N.N.); soon.lee@westernsydney.edu.au (C.S.L.); 2 Department of Anatomical Pathology, Liverpool Hospital, Liverpool 2170, Australia; 3 Cancer Pathology Laboratory, Ingham Institute for Applied Medical Research, Liverpool 2170, Australia; 4 CONCERT Biobank, Ingham Institute for Applied Medical Research, Liverpool 2170, Australia; joseph.po@health.nsw.gov.au; 5 Surgical Innovation Unit, Department of Surgery, Westmead Hospital, Westmead 2145, Australia; 6 Head and Neck Surgery, Liverpool Hospital, Liverpool 2170, Australia; 7 School of Medicine, Western Sydney University and Ingham Institute for Applied Medical Research, Liverpool 2170, Australia; Tara.Roberts@westernsydney.edu.au; 8 South Western Sydney Clinical School, University of New South Wales, Liverpool 2170, Australia; 9 Department of Tissue Pathology and Diagnostic Oncology, Royal Prince Alfred Hospital, Camperdown 2050, Australia; 10 Central Clinical School, University of Sydney, Camperdown 2050, Australia

**Keywords:** thyroid cancer, genomics, immunotherapy, microenvironment, PD-L1

## Abstract

Thyroid cancer is the most prevalent endocrine malignancy that comprises mostly indolent differentiated cancers (DTCs) and less frequently aggressive poorly differentiated (PDTC) or anaplastic cancers (ATCs) with high mortality. Utilisation of next-generation sequencing (NGS) and advanced sequencing data analysis can aid in understanding the multi-step progression model in the development of thyroid cancers and their metastatic potential at a molecular level, promoting a targeted approach to further research and development of targeted treatment options including immunotherapy, especially for the aggressive variants. Tumour initiation and progression in thyroid cancer occurs through constitutional activation of the mitogen-activated protein kinase (MAPK) pathway through mutations in *BRAF*, *RAS*, mutations in the phosphatidylinositol-4,5-bisphosphate 3-kinase (PI3K) pathway and/or receptor tyrosine kinase fusions/translocations, and other genetic aberrations acquired in a stepwise manner. This review provides a summary of the recent genetic aberrations implicated in the development and progression of thyroid cancer and implications for immunotherapy.

## 1. Introduction

Thyroid cancer (TC) is the most common endocrine malignancy, constituting 2.1% of all newly diagnosed cancer cases worldwide [1]. TC incidence has increased 3-fold over the past three decades, with the incidence-based mortality rate increasing at 1.1% per year overall [2]. The incidence is higher in developed countries, and in females with a female to male ratio of 3:1 with a 5.1% incidence in females worldwide in 2018 [2,3]. It was the 7th commonest cancer in women in Australia in 2019 with higher prevalence in 15- to 24-year-olds [4]. Between 1982 and 2019, the age-standardised incidence rate of thyroid cancer increased by 392% (from 2.7 to 13 per 100,000 persons) [4]. This increase is in both genders, all age groups and ethnicities with papillary thyroid carcinomas accounting for the most cases [5,6].

Increased medical surveillance and advent of new radiological diagnostic techniques (low-cost ultrasonography for a more specific thyroid nodule screening), have contributed to increased detection of clinically occult cancers [7]. The increased incidence is attributed to overdetection of small indolent tumours and a concurrent increase in subsequent surgical intervention rates; therefore, patients are overtreated for low-risk tumours [5,7]. The American Thyroid Association treatment guidelines base treatment on risk stratification to prevent overtreatment, and includes assessment of clinical factors (age, gender, radiation exposure and family history), pathological parameters (type and size of tumour, lymphovascular invasion, extrathyroidal extension and lymph node metastases) and molecular markers (including *BRAF*^V600E^, *RAS*, *TP53* and *TERT*) [8].

Importantly, overtreatment may subject patients to possible treatment-related complications without a meaningful improvement in clinical outcomes. Moreover, the cost of interventions in the indolent tumour setting can impose a significant undue financial burden to the healthcare system. Therefore, it is imperative to identify ‘high-risk’ patients to guide appropriate treatment and prevent overtreatment of ‘low-risk’ patients.

## 2. Classification

Thyroid tumours are classified histologically based on criterion implemented by the World Health Organisation (WHO) [9]. The primary tumours are epithelial in origin, developing from the follicular or parafollicular cells. The follicular-derived carcinomas are well differentiated carcinomas (DTC)–papillary carcinomas (PTC), follicular carcinomas (FTC) and Hürthle cell carcinoma (HCC), poorly-differentiated carcinomas (PDTC) and undifferentiated anaplastic carcinomas (ATC). PTCs are diagnosed primarily based on nuclear morphology, and are further subclassified morphologically and architecturally with certain subtypes correlating with an aggressive biology.

DTCs are believed to develop from pre-existing benign and/or borderline follicular neoplasms that display high-risk morphology, notably vascular and capsular invasion, in combination with specific genetic aberrations, whilst the clinically aggressive PDTCs and ATCs develop from dedifferentiation of the DTCs. The borderline/intermediate follicular neoplasms include “follicular tumour of uncertain malignant potential” in which conclusive malignant features are equivocal, and “non-invasive follicular thyroid neoplasm with papillary-like nuclear features (NIFTP)”, an encapsulated circumscribed tumour with stringent histopathologic diagnostic criteria. This criterion constitutes histologically imperative features including well circumscription or encapsulation, PTC nuclear features, a follicular growth pattern and an absence of capsular or vascular invasion. In addition, specific exclusion features including presence of true papillae (>1%) and/or psammomatous calcifications; solid, insular or trabecular growth pattern (>30%); increased mitoses (≥3 mitoses per 10 high-power fields) and tumours necrosis [9]. NIFTP was formerly termed non-invasive encapsulated follicular variant of PTC (EFVPTC); its reclassification initiated by the Endocrine Pathology Society working group through an international multi-institutional study that established preponderance of RAS genetic aberrations (similar to follicular-derived lesions), deficient common PTC *BRAF^V600E^* genetic aberrations and indolent nature of EFVPTC, preventing overdiagnosis and overtreatment of these tumours [10]. Its incidence is variable, lowest in Asian countries at around 1.6%, and highest in Western countries at around 13.3% [11]. Retrospective studies that re-evaluated previously diagnosed EFVPTC cases after reclassification, determined 1.3–40.7% of non-invasive EFVPTC conformed to the histologic criterion for NIFTP [10,12,13]; the variability in numbers attributed to differing prevalence in the Asian and non-Asians studies.

DTCs represent more than 85% of thyroid carcinomas of which PTCs are most prevalent, with a 5-year survival rate of 97.7% in younger age groups with reduction in survival with advancing age. PDTCs and ATCs constitute 5–10% of thyroid cancers with a poor 5-year survival rate of 5%, and develop from dedifferentiation of DTCs [9,14]. The overall improved survival rate in DTCs is attained from implementation of appropriate management plan including surgery, radioactive iodine (RAI) ablation and lifelong thyroid stimulating hormone (TSH) suppressive therapy, and a favourable tumour biology. Currently, administration of radioactive ^131^ I for ablation of residual thyroid tissue is selectively offered to low and intermediate-risk patients with increased serum thyroglobulin level at 6 months post-surgery and following TSH suppressive therapy or with unfavourable prognostic factors [15,16]. Despite this, 20% and 10% of DTC patients develop locoregional recurrences and distant metastases respectively within 10 years, and these are usually RAI-refractory and generally surgically unresectable with overall 3-year survival of less than 50%. These patients are treated with antiangiogenic multitarget tyrosine kinase inhibitors including lenvatinib and sorafenib, and specific inhibitors for BRAF-, MEK- or ALK-mutant tumours [16]. ATCs have an aggressive clinical course, with patient life expectancy of about 6 to 12 months despite the utilisation of systemic therapies such as adjuvant external beam radiation therapy (EBRT), intensity-modulated radiation therapy (IMRT) with radio-sensitising chemotherapy regimens [9,16].

## 3. Thyroid Cancer Pathogenesis

Thyroid cancers, benign and intermediate (NIFTP) entities develop from follicular epithelial cells, and it is theorised that DTCs develop from benign (example follicular adenomas in the case of follicular thyroid carcinomas [FTCs]) and intermediate lesions (NIFTP). PDTCs and ATCs develop from dedifferentiated of DTCs with approximately 10% of PTCs transforming into these forms through different genetic aberrations accumulated in a stepwise manner [17]. Conversely, medullary thyroid cancer (MTC) occurs in the neuroendocrine parafollicular cells (C cells), and is solely induced by *RET* proto-oncogene mutations [17].

## 4. Genetics of Thyroid Cancers

**Figure 1:**
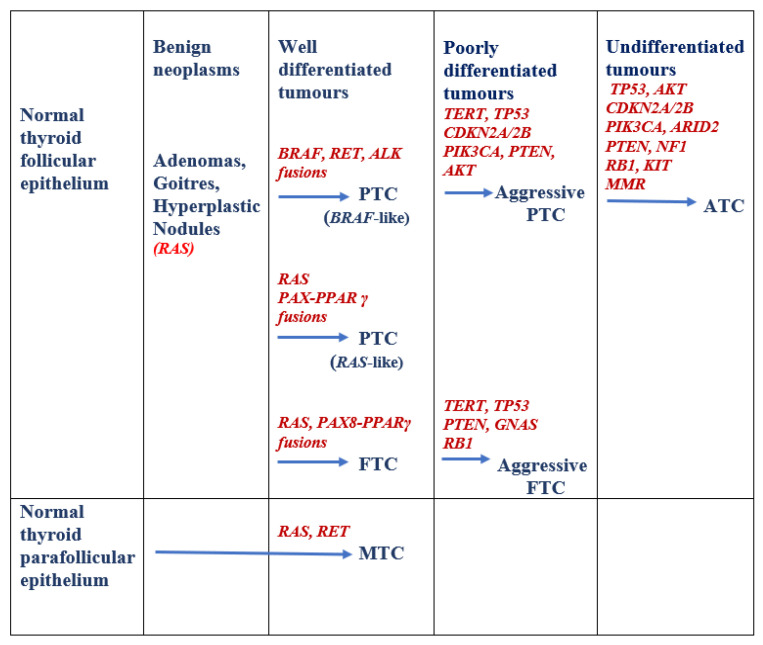
Genetic alterations involved in the evolution of thyroid cancers. Figure adapted from Pozdeyez et al. [18]. Rat sarcoma (RAS). V-raf murine sarcoma viral oncogene homolog B1(BRAF). Rearranged during transfection (RET). Anaplastic lymphoma kinase (ALK). Paired box gene 8-peroxisome proliferator-activated receptor (PAX8-PPARγ). Telomerase reverse transcriptase (TERT). Tumour protein 53 (TP53). Cyclin-dependent kinase inhibitor 2A/2B (CDKN2A/2B). (PIK3CA). Phosphatase and tensin homolog (PTEN). Ak strain transforming (AKT). Guanine nucleotide binding protein, alpha stimulating activity polypeptide (GNAS). Retinoblastoma1 (RB1). AT-rich interactive domain-containing protein 2 (ARID2). Neurofibromatosis1 (NF1). V-kit Hardy-Zuckerman 4 feline sarcoma viral oncogene homolog (KIT). Mismatch repair (MMR). Papillary thyroid carcinoma (PTC). Follicular thyroid carcinoma (FTC). Medullary thyroid carcinoma (MTC). Anaplastic thyroid carcinoma (ATC).

Multiple genes are implicated in the development of thyroid neoplasms, both benign and malignant as illustrated in Figure 1 and Table 1. Specific mutations or rearrangements occur in these genes which are responsible for cell proliferation, survival and differentiation through different pathways. Approximately 90% of mutations are mutually exclusive activating mutations in oncogenes *RAS* (~13%) and *BRAF* (~60%), and rearrangements involving *RET*, *ALK* and *NTRK* genes (~5%); whilst the remaining 10% are loss-of-function mutations affecting tumour suppressor genes such as *PTEN, PPARγ* and *TP53* [17,18,19] Targeted therapies are in use for tumours with some of these mutations. The Cancer Genome Atlas (TCGA) reported comprehensive genetic aberrations in 97% of PTCs, including driver genes *EIF1AX*, *CHEK2* and *PPM1D,* members of the phosphoinositide 3-kinase (PI3K) pathway and other gene fusions [19]. This leaves 3% of PTCs (termed “dark matter”) which remain genetically uncharacterised. Understanding the prevalence and the clinicopathologic relevance of these genetic aberrations have allowed for the pursuance of new targeted therapies.

**Figure 2:**
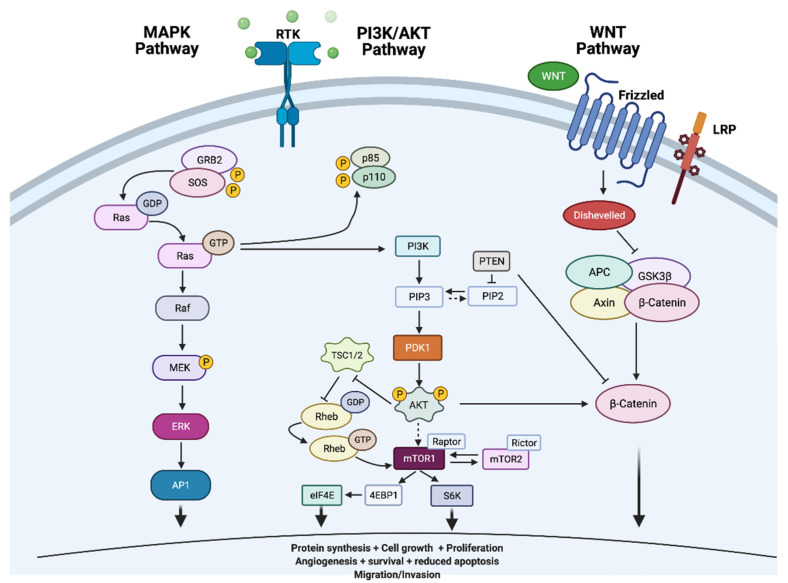
Commonly dysregulated cell signalling mitogen-activated protein kinase (MAPK), phosphatidylinositol-3 kinase (P13K)/Ak strain transforming (AKT) and wingless-related integration site (WNT) pathways in thyroid cancers.

Tumourigenesis of thyroid tumours involves dysregulation of cell signalling pathways mitogen-activated protein kinase (MAPK) and phosphatidylinositol-3 kinase (PI3K)/Ak strain transforming (AKT)/mammalian target of rapamycin (mTOR) signalling pathways. The commonest oncogenic drivers of these pathways include *BRAF* and *RAS* point mutations (Figure 2 adapted from [20] [Created with BioRender.com]).

MAPK and PI3K/AKT pathways are dependent on activity of mutually exclusive *RAS, BRAF* and *RET/PTC* point mutations (BRAF/RAS/PIK3CA) and rearrangements (*RET/PTC* and *TRK*) which drive thyroid cancer oncogenesis. The prevalence of these genetic aberrations varies in the different TCs, with the highest prevalence of up to 62% (most frequently *BRAF^V600E^*) in PTCs [20]. *BRAF^V600E^* mutations are especially found in tall cell variant or infiltrative PTCs which express an aggressive biology, whilst less fatal *RAS* mutations are generally detected in encapsulated and follicular variants of PTCs and adenomas [21,22].

## 5. Tumour Initiation

### 5.1. The Mitogen-Activated Protein Kinase (MAPK) Pathway

The MAP kinase pathway is a signal transduction pathway, constitutive activation of which is essential in the development of TCs. MAPKs regulate vital cellular functions involved in cell proliferation, differentiation and development through key protein including receptor tyrosine kinases (RET, anaplastic lymphoma kinase [ALK], vascular endothelial growth factor receptor [VEGFR] and neurotrophic receptor tyrosine kinase [NTRK1/3]), RAS, rapidly accelerated fibrosarcoma (RAF), mitogen-activated protein kinase (MEK) and extracellular signal-regulated kinase (ERK) as illustrated in Figure 2 below [13]. The binding of a growth factor to a receptor tyrosine kinase receptor activates downstream pathways leading to modification in cell proliferation, differentiation and survival.

The ‘mutual exclusivity’ of mutations and rearrangements has been questioned, which proposes a simultaneous existence of these genes in PTCs that display an aggressive biological behaviour and progress into PDTCs and ATCs in association with additional mutational activators [23,24].

### 5.2. RAS Mutations

The rat sarcoma viral oncogenes homolog (*RAS*) gene is a commonly mutated protooncogene that codes for protein isoforms, *NRAS*, *HRAS* and *KRAS*, which are ubiquitously expressed at different levels in different tissue types*. RAS* proteins act as effector molecules in the MAPK and PI3K/AKT/mTOR signalling cascades and are oncogenically activated in numerous human cancers [25,26]. The *RAS* molecules transmit mitogen signals from the tyrosine kinase membrane receptors (RTKs) to transcription factors via downstream effectors. The *RAS* protooncogenes encode 21kDa G-proteins called p21RAS GTPases that are bound to guanosine diphosphate (GDP) in their inactive form and to guanosine triphosphate (GTP) when active. A group of proteins called ‘GAPs’ (guanine nucleotide exchange factors [GEFs] and GTPase activating proteins) promote conformational changes to the active form by allowing release of GDP, thereby enabling binding of GTP. This conformation change allows transduction of signals from growth factor receptors. Point mutations in the GTP-binding domain (codons 12 and 13) or the GTPase domain (codon 61) cause substitution of certain protein residues that affect GTPase activity, locking p21RAS in the activated form, initiating tumour development. Approximately 99% of *RAS* mutations involve codons 12, 13 or 61 [26,27].

*RAS* initiating mutations inhibit apoptosis promoting transient proliferation of neoplastic epithelial cells in the presence of TSH. This TSH-mediated dependent cell growth is inhibited via programmed cell death through the extracellular signal-regulated kinase (ERK) and c-Jun N-terminal kinases (JNK) signal transduction pathways [28]. Conversely, acute expression of *RAS* in association with absent/low TSH promotes TSH-independent cell growth, highlighting the dependence of thyrocytes expressing oncogenic *RAS* on TSH levels. *RAS* activation causes DNA damage and induces dedifferentiation in a dose-dependent manner with increased *RAS* expression inhibiting thyroid-specific genes that are vital in the maintenance of differentiation (examples transcription factors *TTF-1* and *PAX8*) [28,29,30]. Transgenic mice with human *NRAS* oncogene expression in the thyrocytes under the control of Tg promoter (Tg-NRAS) developed thyroid adenomas (11%) and invasive follicular carcinomas (40%), with approximately 25% showing poor differentiation, lymphovascular invasion and increased metastatic risk [28,30].

Of its three isoforms *NRAS*, *HRAS* and *KRAS*, the most common mutation is the *NRAS* exon2 (codon 61) mutation, which is associated with higher risk of metastasis [31,32,33,34]. *RAS* mutations are documented in both benign and malignant thyroid follicular epithelium with characteristically follicular growth pattern, with higher frequencies in FTC of up to 57%, follicular adenomas 30%, hyperplastic nodules 5.6%, goitres 7–25%, Hürthle cell adenomas 0–4%, NIFTP 29.6–56.6%*)*, and lesser in frequency in PTC of follicular variant with rates of 1.7–20% [10,13,22,32,33,35,36,37]. There is a preferential mutation in the *RAS* subtypes in different thyroid neoplasms with *NRAS* mutations characteristically identified in follicular variants of PTCs, FTCs and ATCs, while Hürthle cell carcinomas (HCC; 15–25%) and medullary thyroid carcinomas (MTCs) commonly harbour *HRAS* mutations [32,38,39]. About 28–55% and 24–52% of PDTCs and ATCs harbour *NRAS*, *HRAS* or *KRAS* mutations, which are mutually exclusive to *BRAF* mutations and *RET*/*PTC* gene fusions [17,40,41].

The development of these tumours is mediated through TSH-independent growth, TSH-dependent apoptosis, DNA damage and de-differentiation, mainly through the *RAF*/*MEK*/*ERK* pathway, stress-activated protein kinase (SAPK)/JNK pathway (apoptosis), and unknown pathway/s involved in RAS-induced dedifferentiation through inhibition of TTF-1 and/or PAX-8. Clinically, the mutational status of *RAS* proto-oncogene is valuable particularly in RAI-refractory thyroid tumours due to advent of MAPK kinase *MEK1* and *MEK2* inhibitor selumetinib [42].

### 5.3. BRAF Mutations

*RAF* is a serine-threonine kinase with three isoforms—*ARAF*, *BRAF* and *CRAF* (*RAF1*). These isoforms are differentially activated by *RAS*, initiating downstream activation of MAPK pathway effectors [43]. The BRAF isoform has the highest affinity for both *MEK1* and *MEK2* [44]. *BRAF* is an initiator mutation, with common valine-to-glutamate substitution at residue 600 (V600E) detected in about 60% of PTCs and is less prevalent in PDTCs (12–33% prevalence) and ATCs (25–29%) [20,45,46,47,48,49,50,51]. These *BRAF*-mutated PDTCs and ATCs arise from pre-existing PTCs. Infrequent *BRAF*^K601E^ mutation, *BRAF* rearrangement and deletions or in-frame insertions represent 1–2% of the remaining cases. The rearrangement occurs through paracentric inversion of chromosome 7q that results in in-frame fusion between exons 1–8 of *AKAP9* gene and exons 9–18 of *BRAF*. This *AKAP9*-*BRAF* rearrangement induces kinase activity, and exists in 11% of PTCs related to radiation exposure and only 1% of those unrelated to radiation exposure [52].

*BRAF* mutations are an early event in tumourigenesis with a presence in papillary microcarcinomas and genotype heterogeneity in TCs [24,53,54]. *BRAF*^V600E^ mutated PTCs have classic papillary morphology or the more aggressive tall-cell morphology [53]. These mutated tumours exhibit reduced expression of genes involved in thyroid hormone biosynthesis, namely thyroglobulin, thyroid peroxidase and sodium iodide symporter, and are refractory to RAI therapy [54,55,56]. *BRAF*^V600E^-mutated tumours typically demonstrate extrathyroidal extension and are generally of advanced stage with *TERT* promoter mutation with evidence of lymph node and distant metastases [53,57,58,59].

*BRAF* activation induces apoptosis, dedifferentiation and promotes TSH-dependent cell growth, however, a resultant balance in apoptosis and synthesis results in no net growth. Through induction of genomic instability, there is acquisition of secondary genetic events that decrease the expression of TSH receptor (TSHR) [60]. Clinically, the higher affinity of *BRAF* for *MEK* suggests utilisation of selective *MEK* inhibitors, such as dabrafenib and trametinib, in addition to *BRAF* inhibitors to preferentially inhibit MAPK pathway-driven growth of *BRAF*-mutated thyrocytes [45,46,58,60,61].

### 5.4. RET/PTC Rearrangements

*RET*, a proto-oncogene located on chromosome 10q11.2 encodes a transmembrane tyrosine kinase receptor which is normally expressed in neural crest-derived cells, including thyroid parafollicular (‘C’) and follicular cells. The *RET* gene is activated by fusion with 5′ portion of heterogeneous genes, initiating the expression of the 3′ portion of the *RET* gene that codes for the tyrosine kinase domain of the receptor, producing constitutively active chimeric forms of the receptor. At least 10 rearrangements with different partner genes are known, and the commonest are *RET*/*PTC1* (formed through paracentric inversion of chromosome 10 long arm which fuses with *CCDC6*/*H4* gene) [62], *RET*/*PTC2* (formed by a reciprocal translocation between chromosomes 10 and 17, leading to juxtaposition of c-RET tyrosine kinase domain with a regulatory subunit of R1acAMP-dependent protein kinase A) [63] and *RET*/*PTC3* (results from fusion with *NCOA4*/*RFG*/*ELE1* gene) [64]. *RET*/*PTC1* and *RET*/*PTC3* rearrangements account for >90% of all rearrangements [65] and occur in higher frequencies in younger patients and those exposed to radiation [66,67,68,69]. These rearrangements activate both the MAPK and PI3K/AKT pathways.

*RET*-*PTC* rearrangement is present in 6.8–32.9% of PTCs and 12.9% of PDTCs [69,70,71,72,73]. *RET*/*PTC* expressing PTCs can be divided into four groups—(1) lacking *RET*/*PTC* rearrangements (28%), (2) balanced *RET* expression with very low levels of *RET*/*PTC1* (24%), (3) unbalanced *RET* exons 10–11 and 12–13 expression with high *RET*/*PTC1* but no *RET*/*PTC3* expression (28%), and (4) unbalanced *RET* expression with high *RET*/*PTC1* and low *RET*/*PTC3* expression (20%) [74]. These rearrangements are associated with distinct tumour biologic properties, with *RET*/*PTC1* tumours displaying the typical papillary architecture, small size and better prognosis; by contrast, *RET*/*PTC3* tumours are solid and aggressive [75]. *RET* rearrangements can occur sporadically in follicular adenomas with a prevalence of 17–63.2% by RT-PCR in Hashimoto’s thyroiditis especially in the metaplastic oxyphil cells [67]. In benign thyroid nodules bearing *RET*/*PTC* rearrangements, there is a 4.3-fold increase in size within a timeframe of about 36 months [76]. This cellular proliferation is through the MAPK pathway and/or through expression of chemokines CXCL1 and CXCL10 and their receptors that modulate cellular growth by an autocrine/paracrine mechanism [77,78]. *RET*/*PTC* activation-linked biological events include apoptosis and dedifferentiation and lack of TSH-independent growth.

The existence of *RET*/*PTC* rearrangements in thyroiditis potentially govern early tumourigenesis, highlighting the role of proinflammatory markers in the development of tumours. Furthermore, the existence of *RET*/*PTC* rearrangements in benign lesions in variable frequencies suggests this to be an initial event. Increased prevalence of *RET*/*PTC* rearrangements in PTCs, with a relatively low prevalence in PDTCs contradicts a potential role of these rearrangements in tumour progression. Therefore, it is implied that additional genetic aberrations occur for progression into aggressive carcinomas. Selpercatinib is a highly selective *RET* kinase inhibitor utilised in patients with *RET* fusion-positive PTC and *RET*-mutant MTC [79].

### 5.5. EIF1AX Mutations

Eukaryotic translation initiation factor 1A X-linked (*EIF1AX*) encodes a translation initiation factor, change-of-function or gain-of-function mutations (in exons 2, 5 and 6) of which were recognised originally in uveal melanomas. These mutations occur in a mutually exclusive with other driver mutations in both benign and malignant TCs including FTCs (17%), HCCs (11%) and PTCs (1–2%; commonly follicular variant). Conversely, 11% of PDTCs and 9–30% of ATCs also harbour these mutations, and are almost consistently associated with *RAS* and *BRAF* mutations [17,19,38,80,81,82]. *EIF1AX* and *RAS* mutations cooperate to drive thyroid tumourigenesis [82]. Co-expression with *RAS* mutations occurs in advanced tumours, which coharbour *TERT* promoter or *TP53* mutations, and are present in approximately 50% of ATCs. *EIF1AX/RAS*-mutated tumours with either *TERT* promoter or *TP53* mutations are larger and aggressive with early metastasis and confer worse survival in PDTCs and ATCs [82]. In thyroid carcinomas, there is a prevalence of hotspot splice-site *EIF1AX*-*A113splice* mutation which initiates eukaryotic initiation factor 2 alpha (EIF2α) suppression by dephosphorylation through induction of activating transcription factor 4 (ATF4; a cellular stress sensor), increasing protein synthesis. *EIF1AX*-*A113splice* also augments cellular myelocytomatosis oncogene (*c-Myc*) stabilisation by *RAS*. *C-MYC* in collaboration with ATF4 induce production of amino acids which sensitise mTOR signalling and protein synthesis [82]. Combinational treatment of mTOR (AZD8055) with either MEK (trametinib) or BRD4 (JQ1) inhibitors to *EIF1AX*-*A113splice* knock-in tumour cell lines result in reduced c-MYC and mTOR protein levels and consequent tumour size reduction [82]. In vivo studies are required to further elucidate the potential use of these inhibitors in humans.

## 6. Tumour Progression

### 6.1. The Phosphatidylinositol 3-Kinase (PI3K)/Ak Strain Transforming (AKT)/Mammalian Target of Rapamycin (mTOR) Pathway

The PI3K/AKT pathway constitutes *PIK3CA*, *PIK3C2G*, *PIK3CGM*, *PIK3C3*, *PIK3R1*, *PRIK3R2*, *AKT1*, *AKT3*, *TSC1*, *TSC2*, *PTEN* and the mTOR signalling complex proteins, alterations of which exist in diverse human malignancies. PI3K/AKT pathway is activated by binding of RAS to the p110 catalytic subunits of PI3K of which PIK3CA (α-type) and PIK3CB (β-type) are the most frequently expressed subunits in tissues. The other common mechanism of activation is through activation of receptor tyrosine kinases by numerous growth factors, leading to activation of the p110 catalytic subunits(s), formation of phosphatidylinositol-3, 4, 5-triphosphate (PIP_3_) which localises AKT to the cell membrane. Phosphorylation of AKT initiates a downstream activation of protein effectors including the mammalian target of rapamycin (mTOR). Phosphatase and tensin homolog deleted on chromosome ten (PTEN) is a key negative regulator of the pathway, a role achieved through dephosphorylation of PIP_3_. Activating mutations or amplification of one of the protein genes, generally *PIK3CA* gene, and/or inactivating mutations (*PTEN*) result in constitutive activation of the pathway, a feature distinguishable in less differentiated tumours [34,83,84,85].

PI3K/AKT pathway’s role in development of thyroid cancer is explained through *PTEN* (the pathway regulator and a major tumour suppressor) germline mutations which notably exist in Cowden disease, a disease characterised by hamartomatous growths, benign thyroid diseases and development of cancers in numerous organs including thyroid [34]. Loss of heterozygosity in *PTEN* occurs in 7% of follicular adenomas and 27% of follicular carcinomas [85,86]. The role of sporadic *PTEN* mutations in thyroid neoplasms is undetermined.

Activation of PI3K/AKT pathway through *PTEN* and/or *PIK3CA* mutation(s) lead to the development of carcinomas especially in the presence of *BRAF*^V600E^ mutation [18,87,88]. *PIK3CA* mutations are prevalent in 5–25% of ATCs and 0–11% in PDTCs; *AKT1* mutations in 0–8% of ATCs and 0–13% of PDTCs; and *PTEN* mutations in 10–15% of ATCs. The difference in mutations across thyroid subtypes is attributed to tumour heterogeneity or impurity due to high macrophage infiltration in ATCs [17,89]. These mutations are uncommon in DTCs, with an 11% prevalence of mutated *PIK3CA* in FTCs, and 3% *PIK3CA* and 2% *PTEN* mutations in PTCs [90]. They are mutually exclusive with *BRAF* or *RAS* mutations in DTCs, whilst occur in combination in PDTCs and ATCs [34]. This observation supports that accumulation of genetic alterations in the PI3K/AKT pathway that potentiates progression of DTCs into PDTCs and ATCs.

Inhibitors of certain PI3K/AKT pathway effectors have been developed, and could be utilised in combination with mainstream chemotherapeutic agents for treatment of thyroid cancers.

### 6.2. PAX8/PPARγ, ALK and NTRK Rearrangements

This rearrangement involves fusion of thyroid-specific transcription factor *PAX8* gene (on chromosome 3p25) with *PPARγ* gene (on chromosome 2q13) [91]. *PPARγ* gene is a transcription factor ubiquitously expressed in adipocytes and functions in lipid metabolism and regulation of adipocyte differentiation [92]. In thyroid neoplasms, *PAX8*/*PPARγ* rearrangement is present in 4–33% of follicular adenomas, 30–58% of FTCs, 0.3% HCC and 37.5% of PTCs of follicular variant [19,32,91,92,93,94].

*ALK* rearrangement is common in PDTCs with a prevalence of 16%; striatin (*STRN*)-*ALK* rearrangement is the commonest and rarely EMAP like 4 (*EML4*)-*ALK* [95,96]. Neurotrophic tyrosine kinase receptor (*NTRK*) fusion oncogenes (*NTRK3*/*ETV6*, *NTRK1*-*TPR* and *NTRK1*-*LMNA* and *NTRK1*-*TMP3*) are recognised in up to 26% of PTCs. NTRK gene fusion positive advanced solid tumours with no conventional treatments are treated with entrectinib or larotrectinib [61,97].

### 6.3. Telomerase Reverse Transcriptase (TERT) Promoter Mutations

Telomerase is expressed in germline cells and its activation promotes cancer development. Activation of telomerase reverse transcriptase (TERT), the catalytic protein subunit of telomerase, is induced by mutations in its promoter region, namely C250T and C228T, the latter being more prevalent, which promotes tumourigenesis by enabling replicative immortality in tumour cells [18]. These mutations co-exist with *BRAF*^V600E^ or *RAS* mutations and are associated with aggressive phenotype with higher invasive and metastatic capability, treatment resistance and poor survival [17,18,98,99,100,101,102]. TERT is overexpressed by binding of *BRAF*^V600E^-induced E26 transformation-specific/E-twenty-six (ETS) transcription factors to the ETS-binding site produced by the mutation. TERT-promoter mutations occur in 9–10.6% non-metastatic PTCs, but their incidence increase incredibly to 60% in metastatic PTCs [18]. These tumours have aggressive clinicopathological features including extrathyroidal extension and lymph node and/or distant metastases [19,103,104]. Genetic analysis studies found the prevalence of clonal TERT promoter mutations in 61–73% of ATCs and 40% of PDTCs, lending further credence to its importance in tumour progression [17]. Recently, TERT rearrangements, which are mutually exclusive with TERT promoter mutations, have been identified in some aggressive cancers such as glioblastoma, neuroblastoma and melanoma as late driver mutations [105,106,107,108]. This finding highlights the important role that TERT promoter mutations play as a late driver in tumour progression in ATCs. Implementation of molecular diagnostic assessment of these mutations could assist in the identification of high-risk patients.

### 6.4. TP53 Mutations

Inactivating point mutations of the tumour protein p53 (*TP53*) tumour suppressor gene that encode p53 protein are prevalent in human cancers including thyroid [109]. Of the thyroid cancers, p53 inactivation is particularly common in PDTCs and ATCs, with a prevalence of 8–35% in PDTCs and significantly higher prevalence of up to 73% in ATCs, especially in association with *BRAF*^V600E^ mutation [17,19,20]. *TP53* alterations are infrequent in metastatic PTCs (13%) or FTC (8%) [109]. This implies that p53 inactivation in association with other oncogenes, for example *BRAF*^V600E^, induces a malignant phenotype in thyrocytes with loss of differentiation and, therefore, is crucial in late-stage progression of thyroid cancer. This knowledge has been utilised in transgenic mouse studies, one of which demonstrated *BRAF*^V600E^ and *TP53*-mutant mice harboured PTCs with accelerated growth and poorer prognostic features including progression into less differentiated PTDCs and ATCs [110]. The latter findings have also been replicated in humans [111,112]. TP53 is a late driver mutation.

### 6.5. CDKN2A

Cyclin dependent kinase inhibitor 2A (*CDKN2A*) encodes p16^INK4a^, a tumour suppressor gene that regulates the cell cycle. Inactivation of the *CDKN2A* gene due to copy number loss (CNL), likely secondary to epigenetic silencing, homozygous loss or truncating mutations is linked to cancer progression. *CDKN2A* mutations in thyroid cancer are mostly associated with advanced status with an incidence of 15–23% in ATCs [18]. *CDKN2A* loss is associated with advanced DTCs, has a higher prevalence in ATCs, and is associated with poor survival [18]. Cyclin D-cyclin-dependent kinase 4/6 (CDK4/6) inhibitor palbociclib could potentially be utilised in ATCs [61].

### 6.6. Mismatch Repair Gene Deficiency

Inactivation of DNA mismatch repair gene(s) encoding MutL-homolog DNA mismatch repair (MMR) enzymes MLH1, MLH3, PMS1 and PMS2 results in microsatellite instability. This inactivation is attributed to persistent oxidative stress which also results in genomic damage and inefficient DNA repair. The prevalence of microsatellite instability (MSI) in thyroid neoplasms is exceptionally rare, and is recognised in about 2.3–2.5% FTCs and is absent or exceedingly rare in other thyroid tumours [113,114,115]. However, Pozdeyev et al. noted presence of MMR DNA deficiency in up to 46% of thyroid cancers with high mutational burden especially ATCs, and these tumours lacked *RAS*, *BRAF* or *RET* oncogenes [18]. The mechanism of the *MLH1* DNA mismatch repair (MMR) silencing in PTC is idiopathic, but is likely promoted by *FOXO1* suppression, FTC and FTA retain MMR activity because of its separate tumorigenic pathways [114,115]. Numerous other studies have produced conflicting results. Santos et al. and Mitmaker et al. documented rates of 37–90% in benign lesions, 64–84% in PTCs and 62.5–87% in FTCs [116,117]. In contrast, other studies documented an almost complete absence of MSI [118,119]. The disparate results are likely attributed to false positives from the use of polyacrylamide gels, whist recent use of next generation sequencing support low prevalence of MSI. Further studies are required to understand the role of mismatch repair gene deficiency in thyroid neoplasms as they respond to anti-PD-L1 (programmed cell death 1 ligand 1) immunotherapy [120].

### 6.7. SWI/SNF Chromatin Remodelling Complex

SWI/SNF complexes consist of 12–15 subunits including ARID1A, ARID1B, ARID2, ARID5B, SMARCB1, SMARCA4, SMARCA2, PBRM1 and ATRX. These complexes interact with co-activators, co-repressors and transcription factors to mobilise nucleosomes, remodel chromatin and repair DNA. The transcriptional regulation role includes development of numerous cell lineages including T-cells and neural cells. An estimated 20% of all human tumours harbour mutations in these genes. Most of the mutations lead to loss of function of the affected tumour suppressor protein with studies highlighting that there is oncogenic activation of the residual complexes, promoting growth by possibly acquiring gain of function and subsequent gene expression facilitating transformation. Loss-of-function mutations in even one of the subunits are identified in 6% of PDTCs and 36% of ATCs [17,121,122].

### 6.8. Wnt Signalling Pathway

This pathway includes proteins encoded by *CTNNB1*(β-catenin), *AXIN1* and *APC* genes that function in cell adhesion and transcription. β-catenin is an essential component of the Wnt signalling pathway, which is activated in numerous tumours [123,124]. APC binds β-catenin and recruits casein kinase I and glycogen synthase kinase-3 (GSK3) that phosphorylate β-catenin for subsequent ubiquitination and degradation by proteasome. This allows constitutive downregulation of β-catenin. β-catenin degradation is inhibited by the Wnt signalling pathway, a process achieved through inhibition of β-catenin phosphorylation, thus permitting translocation of β-catenin to the nucleus to function as a transcription factor. This process is enhanced through blockage of β-catenin binding to cadherin, overactivity of the Wnt pathway or defect in the GSK3β-axin-APC mediated degradation of β-catenin (secondary to mutations in *APC* or *CTNNB1* genes) [125,126]. Additionally, TERT positively regulates this pathway, leading to its activation and resistance to antigrowth signals with subsequent cellular proliferation. Approximately 25% and 65% of PDTCs and ATCs contain these aberrations, with evident nuclear localisation of β-catenin, an infrequent process in DTCs [127]. Furthermore, E-cadherin loss is prominent in PDTCs and ATCs [128,129]. Therefore, β-catenin mutations and loss of E-cadherin expression initiate the tumour dedifferentiation process and further progression.

### 6.9. Epigenetic Modifications in Thyroid Neoplasms

Epigenetic modifications include DNA methylation and histone deacetylation, which regulate gene expression. DNA methylation is a covalent modification of cytosine residues that are present at the dinucleotide sequence CpG, which if unmethylated lead to increased gene transcription, and in contrast hypermethylation of vital gene promoter regions result in heritable inhibition of gene transcription [130].

In thyroid neoplasms, aberrant methylation of thyroid-specific tumour suppressor genes drives dedifferentiation and occurs in the initial phase of tumourigenesis. Reduced or absent TSH-promoted iodine uptake is linked to silencing of thyroid-stimulating hormone receptor (*TSHR*) expression. Silencing of the *TSHR* gene is secondary to hypermethylation of the *TSHR* promoter and is identified in 59–87% of PTCs and 47–50% of FTCs, and PTCs with metastasis [131,132,133]. Methylation is absent in normal human thyroid cells and adenomas. The silenced cell lines if treated with a demethylating agent can partially restore TSHR expression and subsequent TSH-promoted iodine uptake, permitting the use of and enhancing the effectiveness of RAI when used in conjunction with a demethylating agent. Kim et al. showed 90% reduction in thyroid hormone receptor β (*THRB*) mRNA expression in differentiated thyroid carcinomas, in particularly those with advanced histologic features suggesting an inverse correlation, which when treated with demethylating agents 5′-aza-2′ deoxynucleotide and/or zebularine induced 5.6 fold increase in re-expression of the *THRB* gene and concurrent inhibition of tumour growth by inhibition of cell proliferation and migration through the suppression of β-catenin signalling pathway [133]. Silencing of this gene (*THRB* or *THRA*) through promoter hypermethylation is recognised in lung, breast, colon and lymphoid tumours [134,135,136,137,138].

In addition to *TSHR*, numerous tumour suppressor genes silenced through aberrant methylation include genes encoding cyclin-dependent kinase inhibitors p15INKa and p16INK4b [139], RASSF1A [140], RARβ-2(retinoic acid receptor β-2), ECAD, NIS-I, ATM, DAPK (death-associated protein kinase), TIMP3 (tissue inhibitor of metalloproteinase-3), SLC26A and SLC5A8 (sodium monocarboxylate transporter) [141,142]; the latter four are associated with aggressive features.

### 6.10. Copy Number Variations

TCGA discovered presence of somatic copy number variations in 27.2% of TCs deficient of fusions or driver mutations [19]. These variations are variably common in invasive FVPTCs and tall cell variant PTCs. The number of variations correlate with tumour differentiation, with reduced prevalence in well-differentiated tumours (up to 27.1%). The common variations include loses in 22q (includes NF2 and CHEK2) and 10q, and gains in 1q and oncogenic drivers BRAF (in BRAF wild-type tumours) and TERT. Chromosome 1q and TERT amplifications are correlated with aggressive tumour biology [19,143]. ATCs harboured significantly higher genetic variations and tumour mutational burden, with no correlation with age. Conversely, PTCs gain genetic variations with increasing age [18].

### 6.11. Other Genetic Aberrations–Mitochondrial DNA and Genomic Haploidisation

Mitochondrial DNA (mtDNA) incorporate16,569 base pairs and encodes 13 constituents of the apparatus of cellular energy production or the electron transport chain, 2 RNA classes and 22 mitochondrial transfer RNAs. mtDNA has a 10–20 times more mutational rate than nuclear DNA, secondary to oxidative stress. One of these mutations characteristically seen in Hürthle cell tumours is a deletion of 4977 bp called “common deletion,” [144]. Complex 1 mtDNA mutations (loss of function and missense) affect 60% of HCCs [145].

Gopal et al. identified a near-haploid chromosmal content and chromosomal losses that lead to loss of heterozygosity across a large part of the genome in 54% HCCs. In regards, to ploidy, 41.46% were “near-haploid” (mean ploidy <1.6), 46.34% “quiet” (mean ploidy of >1.6 to <2.5) and 12.2% “complex” (mean ploidy >2.5) [145].

Both mtDNA mutations and near-haploid status are important driver events in tumourigenesis of HCCs. These aberrations or their metabolic consequences could be therapeutically targeted, a potential future direction for further research.

**Table 1:** Genomic aberration landscape in benign thyroid lesions and thyroid cancer subtypes.

Genetic Aberration	Benign/Borderline	Follicular Thyroid Carcinoma (FTC)/Hürthle Cell Carcinoma (HCC)	Papillary Thyroid Carcinoma (PTC)	Poorly Differentiated Thyroid Carcinoma (PDTC)	Anaplastic Thyroid Carcinoma (ATC)	Clinical Implication
RAS Point Mutations	28.1–30% (Follicular adenoma) [33,35] 5.6% (Hyperplastic nodule [HN]) [33,35] 7–25% (Goitres [G]) [33,35] 0–4% (Hürthle cell adenoma [HCA])) [32] 29.6–55.6% (Non-invasive follicular thyroid neoplasm with papillary like nuclear features [NIFTP]) [10,13,22]	20–57% (Follicular thyroid carcinoma [FTC]) [33] 15–25% (Hürthle cell carcinoma [HCC]) [32,38]	1.7–52% (follicular variant of papillary thyroid carcinoma [FVPTC] [19,32,36] 13% (classic variant of papillary thyroid carcinoma [CVPTC]) [14]	28–55% [22,40]	23–52% [17,19,40]	Downstream Mitogen-activated protein kinase (MEK)1/2 inhibitor (selumetinib) [42] Higher metastasis risk with N-Rat sarcoma (RAS) codon 61 mutation147 Follicular morphology
V-raf murine sarcoma viral oncogene homolog B1(BRAF) activating mutations (most common is p.V600E; others are p.K601E and small deletions) and fusion (AKAP9-BRAF)	3.7% NIFTP [10]		Up to 62% (mostly CVPTC) [20,45,46,47,48,49,50,51]	12–33% [20,45,46,47,48,49,50,51]	25–29% [20,51]	Selective MEK inhibitors (dabrafenib and trametinib) and BRAF inhibitors (vemurafenib and dabrafenib) Classic and tall cell morphology [20,53,54,55] Refractiveness to radioactive iodine (RAI) [52,53,55] Increased unfavourable prognostic Factors [47,52,53,55]
Rearranged during transfection (RET)-PTC rearrangements	17–63.2% (HT)		6.8–32.9% [69,70,71,72,73]	12.9% [69,70,71,72,73]		Selective RET kinase inhibitors (e.g., selpercatinib) [79]
Eukaryotic translation initiation factor 1A X-(E1F1AX) activating mutations	5–10% (FA) [81,82] 0–5% (HN) [81,82]	17% (FTC) [81,82] 11% (HCC) [38]	1–2% (mostly FVPTC) [18,80,82]	5–15% [17,80,81,82]	9–30% [17,80,81,82]^2^	Co-expression with RAS mutations to drive tumourigenesis [82] Co-expression with tumour portein (TP) 53/Telomerase reverse transcriptase (TERT) mutations in biologically aggressive tumours [82]
Paired box gene 8-peroxisome proliferator-activated receptor (PAX8-PPARγ) rearrangement	4–33% (FA) [19,32,91,92,93,94] 22% NIFTP [10]	30–58% (FTC) [19,32,91,92,93,94] 0–3% (HCC) [38]	37.5% (FVPTC), <1% (CVPTC) [19,32,92,93,94]			Follicular phenotype
TERT promoter		1–35% [19]	9–15% [19]	40% [17]	73% [17]	Usually aggressive biology
TP53		8% [109]	13% [109]	8–35% [17,19,20]	Up to 73% [17,19,20]	Usually aggressive biology
Cyclin-dependent kinase inhibitor 2A/2B (CDKN2A/2B)					15–23% [89]	Aggressive biology. Possible utilisation of Cyclin dependent kinases (CDK) 4/6 inhibitor (palbociclib) [61]
Catenin beta 1 (CTNNB1) activating mutations				Up to 25% [127,128,129]	Up to 65% [127,128,129]	Usually aggressive biology
Anaplastic lymphoma kinase (ALK) fusions (STRN or EML4) or activating mutations			0.8% [20]	Up to 16% [95,96]	0–10% [95,96]	ALK inhibitors
Tyrosine kinase (NTRK)1/3 fusions		0–5% [19]	1.3–26% [19,90]			Targeted therapies (entrectinib or larotrectinib) [61]
Others	Phosphatase and tensin homolog (PTEN) loss of heterozygosity (7% FAs) [85,86] THADA (22% NIFTP) [11]	Phosphatidylinositol-4,5-bisphophate 3-kinase catalytic subunit alpha (PIK3C) (0–11% FTC) [19] PTEN (0–27%) [85,86] Thyroid stimulating hormone receptor (TSHR) BRAF^K601E^ Copy number variations (CNVs) Mismatch repair (MMR) genes mtDNA and diploidies (HCC)	PIK3CA (3%) [19,90] PTEN (2%) [90] BRAF^K601E^ Thyroid adenoma-associated protein (THADA) (5%) [19] TSHR MMR genes [115,116] Copy Number variations (CNVs)	PIK3CA (0–11%) [17,90] PTEN (5–20%) [17,89] Ak strain transforming (AKT)1 (0–13%) [17,89] Switch/sucrose non-fermentable (SWI/SNF) complex subunit mutations CNVs	PIK3CA (5–25%) [17,89] PTEN (10–15%) [17,89] AKT1 (0–8%) [17,89] Ataxia telangiectasia mutated (ATM), retinoblastoma 1 (RB1), Multiple endocrine neoplasia (MEN1) Neurofibromatosis (NF1), NF2, AT-rich interacting domain containing protein 2 (ARID2), MMR genes, V-kit Hardy-Zuckerman 4 feline sarcoma viral oncogene homolog (KIT), SWI-SNF complex subunit mutations CNVs	AKT1 mutation is present in metastatic or recurrent RAI-refractory tumours [89]

## 7. Tumour Microenvironment and Programmed Cell Death 1 Ligand 1 (PD-L1) Expression

The tumour microenvironment (TME) in thyroid cancer plays a crucial role in tumour progression and metastasis. Its constituent immune cells include regulatory T-cells/Tregs, cytotoxic CD8^+^ T-cells, helper T-cells, tumour-associated macrophages, mast cells, natural killer cells, dendritic cells and cytokines [146].

CD4^+^Foxp3^+^CD25^+^ Tregs are exclusively enriched in the TME through their recruitment by cytokines/chemokines and certain growth factors, namely vascular endothelial growth factor and transforming growth factor-β, exerting an anti-tumour immune response and tumour evasion [146,147]. These specific T-regs are associated with aggressive clinicopathological features in TCs, and are notably less potent in benign conditions (e.g., multinodular goitre and Hashimoto’s thyroiditis) [148,149]. Tumour cells express ligands that are recognised by CD8^+^ T-cell receptors, which can be evaded to allow tumour death. Two subsets of tumours are recognised—(i) T-cell-inflamed phenotype consisting of tumour infiltrating T-cells, chemokines and interferon-1, and (ii) non-T-cell-inflamed phenotype [146]. The former is associated with BRAF and RET/PTC positive TCs. Macrophages and other myeloid cells produce cytokines that assist in tumour immune evasion. Tumour-promoting M2 macrophages are induced in tumours by specific cytokines including interleukin-4 (IL-4), IL-10, IL-13 and macrophage colony-stimulating factor (M-CSF). These M2 macrophages produce IL-10, IL-13 and tumour growth factor-β (TGF-β) which suppress immune response, promoting tumourigenesis; and also produce CCL22 that recruits CD4^+^Foxp3^+^CD25^+^ Treg cells. Tregs suppress immune system to maintain homeostasis and self-tolerance by inhibiting T-cell proliferation and cytokine production. Studies established high M2 macrophage infiltration in ATCs with expression of about 68 genes associated with M2 macrophages [17,146,150]. Furthermore, tumour infiltrating CD8^+^effector T-cells, including functionally exhausted ones significantly increase with tumour progression with higher quantities detected in PDTCs and ATCs, and are infrequent or absent in PTCs and normal thyroid tissue. Therefore, two immune signature types in thyroid cancer are introduced based on the immune cell infiltration levels: ATC-like and PDTC-like [151].

Upregulation of CD274 (encodes PD-L1), an immune checkpoint protein ligand for programmed cell death-1 (PD-1) has been reported in ATCs. PD-1 is a transmembrane protein which is expressed on immune cells [152]. Expression of PD-L1 on the surface of tumour cells allows for engagement with PD-1^+^ T cells, resulting in T cell dysfunction by exhaustion, anergy, apoptosis and IL-10 expression. There is consequential evasion of the host immune-mediated tumour destruction, resulting in tumour spread, relapse and metastasis [153]. PD-L1 expression in DTCs, PDTCs and ATCs is associated with poor survival, especially in co-existence with BRAF^V600E^ mutation [154,155], and is noted in 6.1% PTCs, 7.6% FTCs and 22.2% ATCs [156,157,158]. Monoclonal antibodies targeting both PD-1 and PD-L1 have illustrated reduced tumour growth and increased survival [156,157,158,159]. Recently identified PD-L1 regulatory proteins CMTM4 and CMTM6 are documented to enhance a PD-L1 tumour’s ability to evade the immune system by inhibiting T-cells. Any interference with their expression leads to consequent altered PD-L1 protein expression on tumour cells, suggesting a potential for development of future immunotherapeutic agents. The role of these proteins on the PD-1/PD-L1 axis further needs elucidation [160]. PD-L1 expression is higher in ATCs (65%), and less frequent or absent in DTCs. Role of PD-L1 inhibitors such as pembrolizumab are currently under investigation [161].

The utilisation of PD-1/PD-L1 immunotherapies with multiple kinase inhibitors has proven effective in the treatment of ATC. Gunda et al. documented that Lenvatinib, a multi-targeted tyrosine kinase, in combination with PD-L1 inhibitors promoted tumour size reduction and increased overall survival in murine model. Lenvatinib monotherapy promoted production of CD8^+^ T-cells, T_regs_, tumour infiltrating macrophages and polymorphonuclear myeloid derived suppressor cells (PMN-MDSCs), with increased numbers of the latter postulated in treatment resistance. Combination therapy, also potentially including anti-Gr-1 antibody significantly reduced PMN-MDSCs, enhancing the treatment effect [162]. Human studies are required to evaluate the role of these combined therapies.

Studies are underway to determine the benefits of longitudinal use of PD-1/PD-L1 inhibitors such as cemiplimab with dabrafenib and trametinib, pembrolizumab with lenvatinib, and atezolizumab or spartalizumab with chemotherapy or targeted therapy [159].

A trial underway showed a 20% improvement in tumour size with use of a new *NTRK* inhibitor entrectinib. Tipifarnib (*HRAS* inhibitor) and palbociclib (cyclin D-cyclin-dependent kinase 4/6 inhibitor), both also currently being investigated, have shown reduction in tumour size [163]. Further development in targeted therapy for ATCs is required.

## 8. Summary

Thyroid carcinomas arise and progress through some mutually exclusive genetic aberrations in MAPK and PI3K pathways. De-differentiation of these tumours occurs through additional genetic mutations in different pathways involved in cellular function. Improved knowledge has conclusively revealed the association of these genetic aberrations with specific tumour histology and biological behaviour. This has promoted research in establishing the possibility of utilisation of some of these specific molecular aberrations as putative prognostic markers and also the development of targeted therapies for biologically aggressive tumours.
